# Unlocking the mystery of the hard-to-sequence phage genome: PaP1 methylome and bacterial immunity

**DOI:** 10.1186/1471-2164-15-803

**Published:** 2014-09-19

**Authors:** Shuguang Lu, Shuai Le, Yinling Tan, Ming Li, Chang Liu, Kebin Zhang, Jianjun Huang, Haimei Chen, Xiancai Rao, Junmin Zhu, Lingyun Zou, Qingshan Ni, Shu Li, Jing Wang, Xiaolin Jin, Qiwen Hu, Xinyue Yao, Xia Zhao, Lin Zhang, Guangtao Huang, Fuquan Hu

**Affiliations:** Department of Microbiology, College of Basic Medical Science, Third Military Medical University, Chongqing, 400038 P. R. China; IMPLAD/PacBio joint laboratory for advanced genomic analysis, Institute of Medicinal Plant Development, Chinese Academy of Medical Sciences, Peking Union Medical College, Beijing, 100193 P. R. China

## Abstract

**Background:**

Whole-genome sequencing is an important method to understand the genetic information, gene function, biological characteristics and survival mechanisms of organisms. Sequencing large genomes is very simple at present. However, we encountered a hard-to-sequence genome of *Pseudomonas aeruginosa* phage PaP1. Shotgun sequencing method failed to complete the sequence of this genome.

**Results:**

After persevering for 10 years and going over three generations of sequencing techniques, we successfully completed the sequence of the PaP1 genome with a length of 91,715 bp. Single-molecule real-time sequencing results revealed that this genome contains 51 *N*-6-methyladenines and 152 *N*-4-methylcytosines. Three significant modified sequence motifs were predicted, but not all of the sites found in the genome were methylated in these motifs. Further investigations revealed a novel immune mechanism of bacteria, in which host bacteria can recognise and repel modified bases containing inserts in a large scale. This mechanism could be accounted for the failure of the shotgun method in PaP1 genome sequencing. This problem was resolved using the *nfi*^-^ mutant of *Escherichia coli* DH5α as a host bacterium to construct a shotgun library.

**Conclusions:**

This work provided insights into the hard-to-sequence phage PaP1 genome and discovered a new mechanism of bacterial immunity. The methylome of phage PaP1 is responsible for the failure of shotgun sequencing and for bacterial immunity mediated by enzyme Endo V activity; this methylome also provides a valuable resource for future studies on PaP1 genome replication and modification, as well as on gene regulation and host interaction.

**Electronic supplementary material:**

The online version of this article (doi:10.1186/1471-2164-15-803) contains supplementary material, which is available to authorized users.

## Background

Whole-genome sequencing is a very important method to understand the genotype and phenotype of an organism. In 1976, the genome of phage MS2 (only 3.5 kb in length) was the first completely sequenced genome [1]. The whole genome sequence of phage φX174 (with 5.3 kb genome) was then reported a year later [2]. Early genome-sequencing studies mainly focused on small genomes. With the advancement of sequencing technologies, particularly shotgun sequencing method [3, 4], the sequencing of large genomes has become possible. Thus far, next- and third-generation sequencing technologies have become available [5–8]. Hence, genome sequencing has shown remarkable development.

However, small genomes, particularly bacteriophage genomes, are occasionally hard to be sequenced. We once encountered a tough work in sequencing a phage genome with a size of approximately 90 kb. In 2004, we isolated and characterised a *Pseudomonas aeruginosa* phage named PaP1 [9, 10]. Pulsed-field gel electrophoresis (PFGE) results showed that PaP1 contains a genome of approximately 90 kb, but 20 contigs obtained using the shotgun library sequencing method could not be assembled in an integral genome; the total length of these obtained contigs was approximately 47.7 kb, which is almost half of 90 kb. We subsequently submitted the PaP1 genomic DNA to another sequencing center, where this DNA was subjected to repeated sequencing with the shotgun method. We obtained almost the same result. We further verified this result by obtaining the PaP1 genome sequence with primer walking [11]; however, we failed again. Hence, this work was suspended.

Four years later, Roche/454 technique [12, 13], a second-generation sequencing method, was established. We re-sequenced the PaP1 genome by using the Roche/454 technique in 2008. We easily obtained the complete PaP1 genome sequence with a size of 91,715 bp. Thus, we aimed to determine why the PaP1 genome was successfully sequenced using the Roche/454 DNA sequencer but not using the shotgun sequencing method. Based on the differences of the principles of the two sequencing methods, our presumption was that the host bacterium of the shotgun library construction, *Escherichia coli* DH5α, may greatly repel the inserted phage-DNA fragments by a particular immune mechanism. In the present study, this hypothesis was confirmed by conducting several experiments, including gene knockout and single-molecule real-time (SMRT) DNA sequencing techniques (third-generation sequencing methods) [6, 14–16]; we also investigated the methylome of phage PaP1. We revealed a novel mechanism of bacterial immunity that could repel exogenous DNA and maintain their genetic stability via enzyme Endo V activity.

## Methods

### Bacterial strains, plasmids and growth conditions

The bacterial strains and plasmids used in this study are listed in Table 1. *P. aeruginosa* and *E. coli* strains were grown in Luria-Bertani (LB) broth and plated onto LB medium containing 1.5% (w/v) agar. Antibiotics were added as needed at the following concentrations: 100 μg/mL ampicillin (Boehringer, Mannheim, Germany) and 25 μg/mL chloramphenicol (Sigma-Aldrich, St. Louis, MO).

**Table 1:** **Bacterial strains and plasmids used in this study**

Strains or plasmids	Relevant characteristics ^a^	Source or reference
Strains		
*Pseudomonas aeruginosa* PA1	Belongs to serum typing group 9 of *P. aeruginosa* international antigenic typing system; host of phage PaP1	Laboratory collection
*P. aeruginosa* PA3	Belongs to serum typing group 6 of *P. aeruginosa* international antigenic typing system; host of phage PaP3	Laboratory collection
*E. coli* DH5α	Host for the construction of shotgun library clones	Promega, WI, USA
*E. coli* DH5α *cat* ^*+*^:Δ*nfi*	The *nfi* gene is replaced with *cat*.	This study
*E. coli* DH5α Δ*nfi*	The *nfi* gene is knocked out.	This study
Plasmids		
pKD3	Template plasmid for Red system; Amp^r^, Cm^r^	[26]
pKD46	Red expression plasmid; Amp^r^	[26]
pCP20	Flp expression plasmid; Amp^r^, Cm^r^	[25]
pUC18	Vector for the construction of shotgun library clones; Amp^r^	TaKaRa, Shiga, Japan

^a^Amp^r^, ampicillin resistant; Cm^r^, chloramphenicol resistant.

### Phage propagation and purification

We isolated PaP1 and PaP3 phages from hospital sewage by using *P. aeruginosa* PA1 and PA3 (Table 1) as host bacteria, respectively, in accordance with standard lambda phage isolation protocol [17]. PaP1 and PaP3 were propagated and purified in accordance with previously described protocols [9, 18, 19] with slight modifications. In brief, the liquid culture of the host bacteria during the log growth phase was inoculated with phages (multiplicity of infection of 1/100) and incubated at 37°C with shaking at 200 rpm. The culture showed signs of lysis after 5 h and a few drops of chloroform were added to ensure that all of the host bacteria were lysed. The culture was then centrifuged at 10,000 × *g* for 5 min; the supernatant (crude PaP1 suspensions) was concentrated and purified via PEG8000 (Sigma-Aldrich, St. Louis, MO) precipitation, as described previously [20]. The PaP1 particles were concentrated using PEG8000 (these particles were placed in an ice bath for 1 h and centrifuged at 12,000 × g for 10 min; the precipitate was then collected) and further purified using a CsCl gradient ultracentrifuge in accordance with previously reported methods [21, 22].

### DNA extraction and purification

EDTA (20 mM), proteinase K (50 μg mL^-1^) and sodium dodecyl sulfate (0.5%, w/v) were added to the purified phage stock solution (PaP1 or PaP3). The mixture was incubated at 56°C for 1 h; an equal volume of phenol-chloroform-isoamyl alcohol solution (25:24:1) was added and the resulting mixture was centrifuged at 5,000 × *g* for 10 min. An aqueous layer was collected and extracted with chloroform at 5,000 × *g* for 10 min. The collected aqueous layer was mixed with 0.6 volumes of isopropanol and stored overnight at -20°C. Afterward, the mixture was centrifuged for 10 min at 12,000 × *g* and 4°C; the precipitated DNA was collected and washed with 70% and 100% ethanol, respectively. The PaP1 DNA was suspended in TE buffer (pH 8.0) and stored at -20°C for subsequent use.

### Endonuclease digestion assay

The following restriction endonucleases were used to digest the genomic DNA of PaP1 or PaP3 in 20 μL reaction systems according to the manufacturer’s instructions: PauI; VspI; AatII; SpeI; and EcoRI (New England Biolabs, Ipswich, MA, USA). The mixture was incubated at 37°C for 120 min and then used to perform PFGE. PFGE was conducted in 1% agarose gel with an initial switch time of 0.6 s and a final switch time of 1.6 s at 8 V/cm and an angle of 180° with a run time of 4.5 h. The restriction map was captured and analysed using Quantity One software (Bio-Rad, Hercules, CA, USA) to estimate the sizes of DNA bands on the gel. The commercial Endo V, or the products of *E. coli* gene *nfi*, was purchased from New England Biolabs, Ipswich, MA, USA. The PaP1 or PaP3 genomic DNA was digested by Endo V in 20 μL reaction systems according to the manufacturer’s instructions.

### Sequencing of the PaP1 genome by using shotgun library method

In 2004, the genomic DNA of PaP1 was submitted to Chinese National Human Genome Center (CNHGC) in Shanghai, China for genome sequencing with the shotgun sequencing method [3] in an ABI 3730 DNA sequencer (ABI, Foster City, CA, USA). A shotgun library was constructed using *E. coli* DH5α as host bacterium. The PaP1 genomic DNA was digested by Sau3AI (New England Biolabs, Ipswich, MA, USA) or treated with ultrasonic waves; the DNA fragments with a length ranging from 1.6 kb to 2.0 kb were recovered to construct the shotgun library. The recovered DNA fragments were ligated into pUC18 and then electrotransformed into the host bacterium *E. coli* DH5α. Clones were selected randomly from the library and used for sequencing. A total of 1,653 clones were sequenced and the average sequence coverage reached approximately 15-fold of the PaP1 genome. The obtained reads were assembled using the Phred/Phrap/Consed software package [23]. We obtained 20 contigs, but these contigs could not be assembled into an integral genome. To obviate mistakes caused by sequencing, we submitted the PaP1 genomic DNA to CNHGC in Beijing, China for repeat sequencing. Although the average sequence coverage also reached approximately 15-fold of the PaP1 genome, the obtained results were almost the same as those of the first sequencing. We also tried primer walking [11] to fill the gaps, but we failed to obtain the whole genome sequence of PaP1.

In 2012, we knocked out the *nfi* gene of *E. coli* DH5α (see below). To validate whether or not the *nfi*^-^ mutant of *E. coli* DH5α can be used to construct a shotgun library and sequence the PaP1 genome, we repeated the sequencing of the PaP1 genome at Genemine Biotechnology Co., Ltd. (Chongqing, China). The procedures were exactly the same as described previously except the shotgun library clones were constructed with the *nfi*^-^ mutant of *E. coli* DH5α as host bacterium. At this time, 1,017 clones were sequenced and the average sequence coverage reached approximately 10-fold of the PaP1 genome.

### Sequencing of the PaP1 genome by using Roche/454 technique

In 2008, next-generation sequencing techniques were established. We then submitted the PaP1 genome to the CNHGC (Shanghai, China) for sequencing with a Roche/454 GS FLX titanium system [12]. In brief, the purified genomic DNA of PaP1 was fragmented, ligated to adapters and separated into single strands; the DNA fragments were bound to beads and amplified by emulsion PCR. A solid-phase pyrophosphate sequencing reaction was performed to reveal the raw sequence data. The Roche/454 reads were assembled using a Newbler assembler [24] (454 Life Sciences). The PaP1 genome sequence and its annotation information were available for download at the NCBI GenBank (http://www.ncbi.nlm.nih.gov/genbank/) with an accession number of HQ832595.

### Construction of the *nfi*^-^mutant of *E. coli*DH5α

The *nfi*^-^ mutant of *E. coli* DH5α was constructed in accordance with previously described protocols [25, 26]. The plasmids used in the procedure are listed in Table 1. The primers and other DNA sequences used in this procedure are listed in Table 2. The primers Cm-F [containing 55 bp upstream homologous extensions of the *nfi* gene (H1)] and Cm-R [containing 55 bp downstream homologous extensions of the *nfi* gene (H2)] were designed using the DNA sequence of pKD3 as a template. The PCR product (donor DNA) that contains the chloramphenicol resistance gene (*cat*) and two FLP (a yeast-derived recombinase) recognition target (FRT) sites were then obtained by two-step PCR with Cm-F and Cm-R primers. The pKD46 plasmid (containing λ-Red recombinase) and the donor DNA were electrotransformed into *E. coli* DH5α. The bacteria were cultured in LB medium containing 100 mM L-arabinose (Sigma-Aldrich, St. Louis, MO) at 30°C for 12 h to induce homologous recombination between *cat* and *nfi* genes. The chloramphenicol-resistant colony was selected and cultured at 42°C for 6 h to eliminate the pKD46 plasmid. The obtained recombination strain was designated as *E. coli* DH5α *cat*^+^:Δ*nfi*. The pCP20 plasmid was electrotransformed into *E. coli* DH5α *cat*^+^:Δ*nfi*; the bacteria were cultured at 42°C for 6 h to induce the FLP recombination of the FRT sites and to eliminate the *cat* gene and the pCP20 plasmid. The final mutant was designated as *E. coli* DH5α Δ*nfi*.

**Table 2:** **Primers and other DNA sequences used in this study**

Primers or other DNA sequences ^a^	Sequence (5′-3′)	Target genes or locations
Construction of the *nfi* mutant		
Cm-F	GTGTAGGCTGGAGCTGCTTC	Chloromycetin-resistant gene of pKD3
Cm-R	CATATGAATATCCTCCTTAG	
Nfi-F	TGTGCCGCCAGAACATGC	*nfi* gene of *E. coli* DH5α
Nfi-R	GACGCAGATGAATTGGGT	
H1	CGTGGAGGCAGTGCATCGACTGTCTGAACAGTATCACCGCTAAGGAGTGATTATG	Upstream of the *nfi* gene
H2	TTTGTAACATGTTGAGTTCTCAAATACGGAAATTATCCGCAGTTTACCTGAATTA	Downstream of the *nfi* gene

^a^Primers and other DNA sequences were synthesised by BGI-Shenzhen (Shenzhen, China).

Nfi-F (upstream of the gene *nfi*) and Nfi-R (downstream of the gene *nfi*) primers were designed to indicate the change in the *nfi* gene. PCR was performed using Nfi-F and Nfi-R primers with the genomic DNAs of *E. coli* DH5α, *E. coli* DH5α *cat*^+^:Δ*nfi* and *E. coli* DH5α Δ*nfi* as templates. The PCR products were used in 0.8% agarose gel electrophoresis (100 V for 40 min) to determine their sizes.

### SMRT sequencing of the PaP1 genome

The PaP1 genome was subjected to SMRT sequencing at the Institute of Medicinal Plant Development (Beijing, China) by using a PacBio RS DNA sequencer (Pacific Biosciences, Menlo Park, CA, USA; http://www.pacificbiosciences.com/) [27, 28]. SMRT sequencing was performed in accordance with previously described protocols [6, 14, 15]. In brief, SMRTbell template libraries with DNA fragments of 2 kb were prepared [29, 30]. Sequencing was then performed using one SMRT cell (http://www.pacificbiosciences.com/products/consumables/SMRT-cells/); zero-mode waveguide (ZMW) [31] signals were obtained. SMRT reads were mapped to the reference sequence of the PaP1 genome by using the BLASR software (https://github.com/PacificBiosciences/blasr) [32] in accordance with standard mapping protocols. Interpulse durations (IPDs) were determined and processed as previously described [15, 29, 33] for all of the pulses aligned to each position in the PaP1 genome sequence. The modified bases were identified using SMRT Analysis Server v. 1.4.0 (Pacific Biosciences). The generated data sets are available for download at the NCBI Gene Expression Omnibus (GEO) (http://www.ncbi.nlm.nih.gov/geo/) [34] with the accession number of GSE50100 [GEO: GSE50100].

### Bioinformatics analyses

DNAStar [35] was used to analyse the basic characteristics of the PaP1 genome sequence. The Internet tool tRNAscan-SE 1.21 [36] was used to predict tRNA genes in the DNA sequence with a cove score cutoff of 20. DNAMAN software (http://www.lynnon.com/) was used to analyse the localisation of the 20 contigs in the PaP1 genome and to graphically describe the result. The PanDaTox database (http://www.weizmann.ac.il/pandatox) [37] was used to analyse the putative DNA motifs that were toxic to bacteria in the PaP1 genome.

The raw modification calls of the PaP1 genomic DNA, produced using the SMRTPortal Analysis Platform v. 1.3.3 (Pacific Biosciences; details are available at http://www.pacb.com/pdf/TN_Detecting_DNA_Base_Modifications.pdf), were collated as single Modifications.gff file. To predict modified motifs, we screened the Modifications.gff file by using publicly available R-scripts software (https://github.com/PacificBiosciences/motif-finding), as well as an online motif finding server (MEME, http://meme.nbcr.net/meme/cgi-bin/meme.cgi) [38]. PaP1 ORF48 was blasted against NCBI non-redundant protein sequences (nr) (http://blast.ncbi.nlm.nih.gov/Blast.cgi?PROGRAM=blastp&PAGE_TYPE=BlastSearch&BLAST_SPEC=&LINK_LOC=blasttab&LAST_PAGE=blastn) to search probable correlations between ORF48 and methyltransferases. Protein sequences were subjected to multiple sequence alignments by using ClustalW [39] with default parameters and a phylogenetic tree was constructed and displayed using MEGA5 [40] with a neighbor-joining method [41].

## Results

### Shotgun strategy failed to obtain a complete PaP1 genome sequence

**Figure 1:**
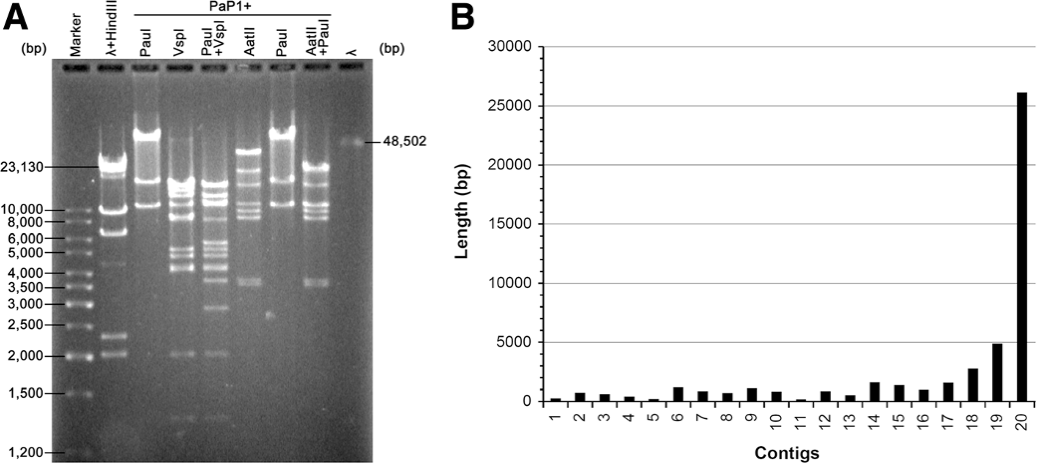
**Shotgun sequencing failed to determine the whole PaP1 genome. (A)** PFGE map of the PaP1 genomic DNA, showing that the PaP1 genome should be approximately 90 kb. **(B)** Length of the 20 contigs obtained using the shotgun method. Contig20 is the longest contig (approximately 26.1 kb) and all of the contigs could not be assembled in an integral genome sequence. The total length of the 20 contigs is approximately 47.7 kb, almost half of 90 kb.

The PFGE result showed that the PaP1 genome is approximately 90 kb in length (Figure 1A). However, the sequencing results of the PaP1 genome by using the shotgun strategy only provided 20 contigs with various lengths (Figure 1B) and all of these 20 contigs could not be assembled in an integral genome. In addition, the overall length of these 20 contigs was approximately 47.7 kb, only almost half of 90 kb. We subjected the PaP1 genome to re-sequencing in another sequencing company by using the shotgun method. However, we obtained almost the same result, as in the first sequencing. We also performed primer walking [11] to fill the gaps, but we still failed to obtain the whole genome sequence of PaP1. Although we selected 216 clones of the random restriction library of the PaP1 genome for sequencing, all of the obtained sequences belong to the sequence sets of the 20 contigs.

### PaP1 genome sequence obtained by Roche/454 sequencer

**Figure 2:**
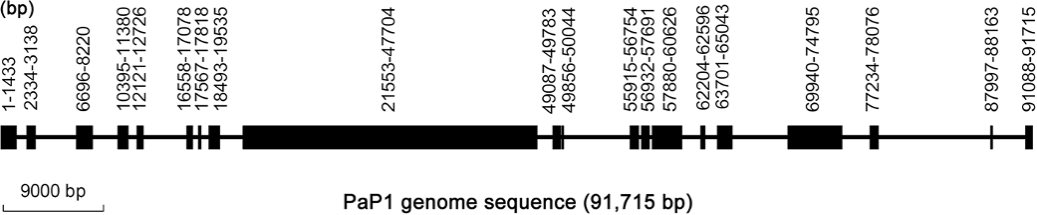
**Distribution of 20 contigs in the PaP1 genome sequence.** The exact location of each contig is shown with a brownish red box. The longest contig (21,553–47,704 position) is approximately 26 kb. The total length of the contigs is approximately 47.7 kb, almost half of 91.7 kb.

Using a Roche/454 DNA sequencer, we easily obtained the 91,715 bp whole genome sequence of PaP1. The PaP1 genome sequence and its annotations have been submitted to GenBank (Accession: HQ832595). On the basis of the comparative analysis results of the PaP1 genome sequence, we established a new genus named PaP1-like phages [9]. The PaP1 genome does not contain complicated secondary structures. To determine the relationship between the sequences obtained by the shotgun method and the Roche/454 DNA sequencer, we mapped the 20 contigs to the PaP1 genome sequence and found that all of the sequences of the 20 contigs are identical to the PaP1 genome sequence; however, gaps with various lengths are present among these contigs (Figure 2). The largest gap was approximately 10 kb, which was very large to be filled by primer walking [11]. The total sequence length of the 20 contigs was approximately 47.7 kb, only half of the whole PaP1 genome sequence (91.7 kb).

### Single-molecule sequencing revealed modified bases in the PaP1 genome

The PaP1 genome could be successfully sequenced with the Roche/454 technique but not with the shotgun method. The shotgun method depends on the construction of a DNA library; by contrast, the Roche/454 technique is a non-library-dependent technique. Therefore, we hypothesised that the shotgun method failed possibly because *E. coli* DH5α, the host bacterium of the shotgun library construction, greatly repelled the inserted DNA fragments by endonucleases; the PaP1 genome may contain modified bases that may be the recognised targets degraded by endonucleases.

**Figure 3:**
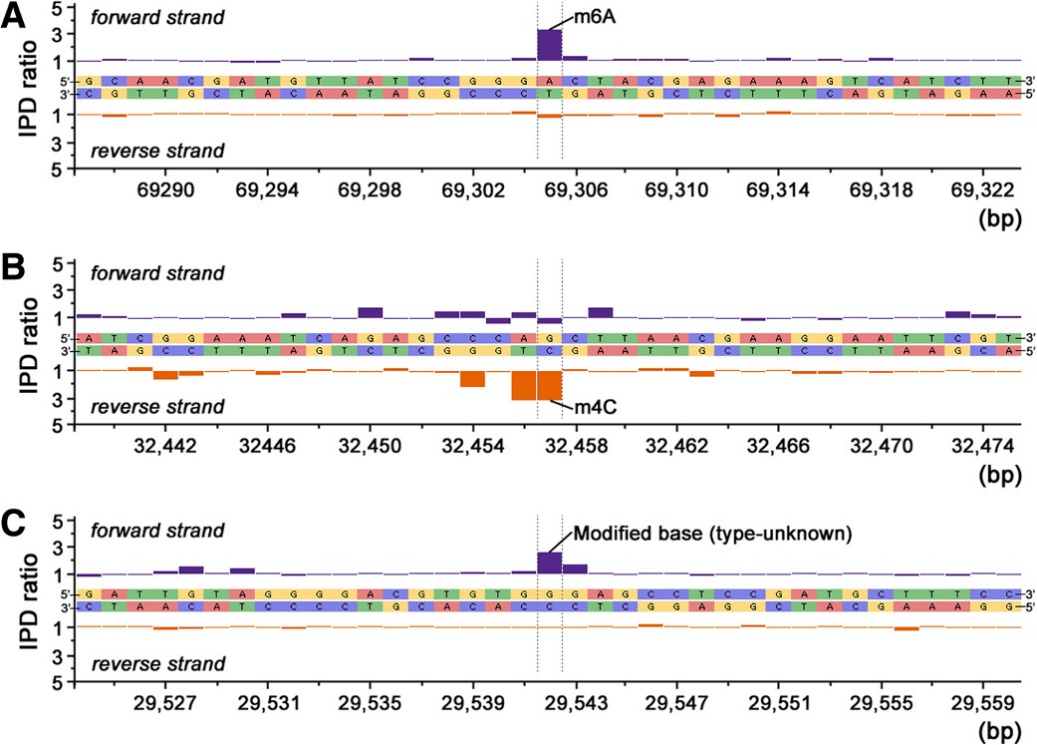
**Trace of IPD ratio variations showing three instances of modified sequence regions in the PaP1 genome. (A)** IPD ratios of an m6A and its surrounding bases. **(B)** IPD ratios of an m4C and its surrounding bases. **(C)** IPD ratios of an unknown modified base and its surrounding bases.

**Figure 4:**
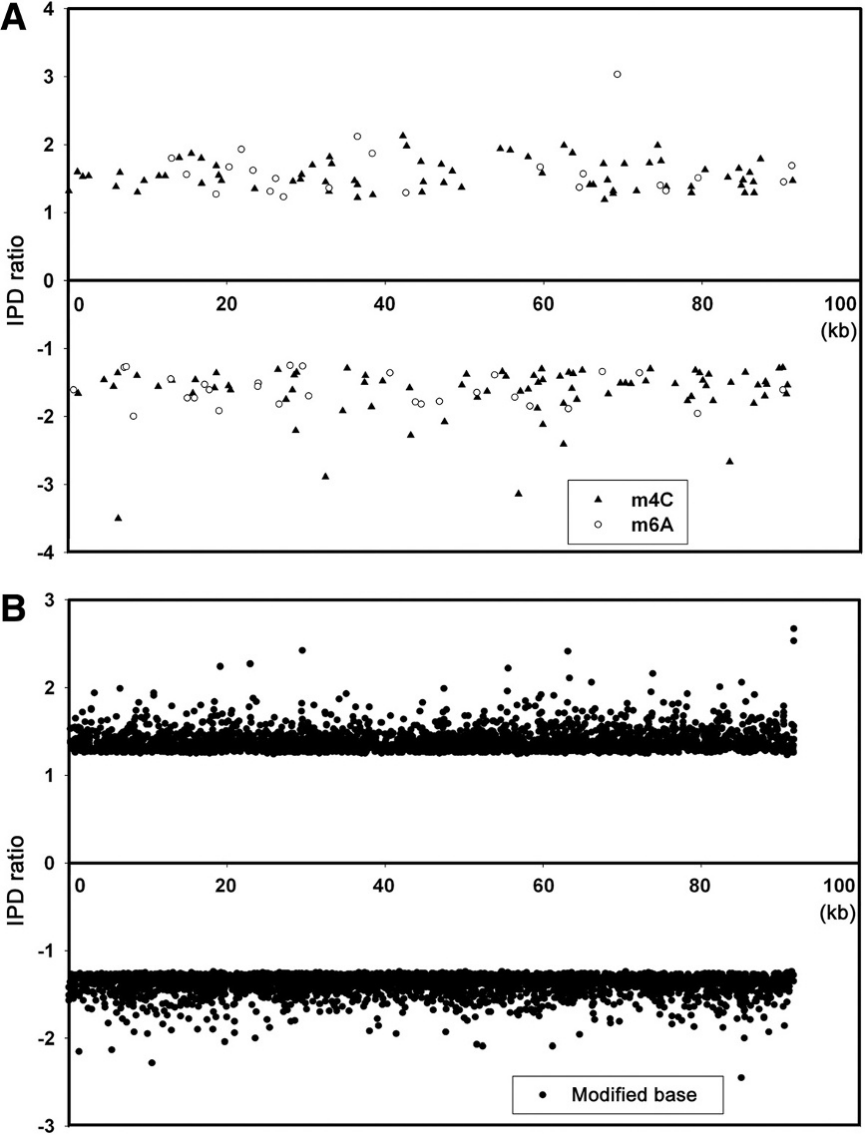
**Distribution of modified bases in the PaP1 genome.** The positive IPD ratios represent the bases on the positive strand and the negative IPD ratios represent the bases on the negative strand. **(A)** The IPD ratios and positions of m4Cs and m6As in the PaP1 genome. **(B)** The IPD ratios and positions of unknown modified bases in the PaP1 genome. See also Additional file 2: Excel S1.

As such, we subjected the PaP1 genome to another sequencing by using a SMRT DNA sequencing technique [15] in 2013. In this procedure, the average sequence coverage of the SMRT sequencing reached 1,380-fold of the PaP1 genome (Additional file 1: Figure S1). We obtained IPD ratios of the 91,715 bases on both positive and reverse strands of the PaP1 genomic DNA. Among the IPD ratios, those of 7,557 bases (Additional file 2: Excel S1) exhibited typical signals of modified bases, including 51 of *N*-6-methyladenines (m6A), 152 of *N*-4-methylcytosines (m4C) and 7,354 other modified bases (unknown modified types because of the limitations of the current SMRT sequencing technique). Figure 3 shows the IPD ratios of both DNA strands in a section of the PaP1 genomic DNA by SMRT sequencing: A, B and C show the three typical instances (m6A, m4C and unknown modified base, respectively) of modified bases. Figure 4 shows an integral epigenetic map of the PaP1 genome, indicating the positions of m6As, m4Cs and unknown modified bases. These results indicated that the PaP1 genome contains numerous modified bases (7,557 in number), accounting for 8.2% of the total PaP1 genome sequence.

### Methylome analysis of the PaP1 phage

**Figure 5:**
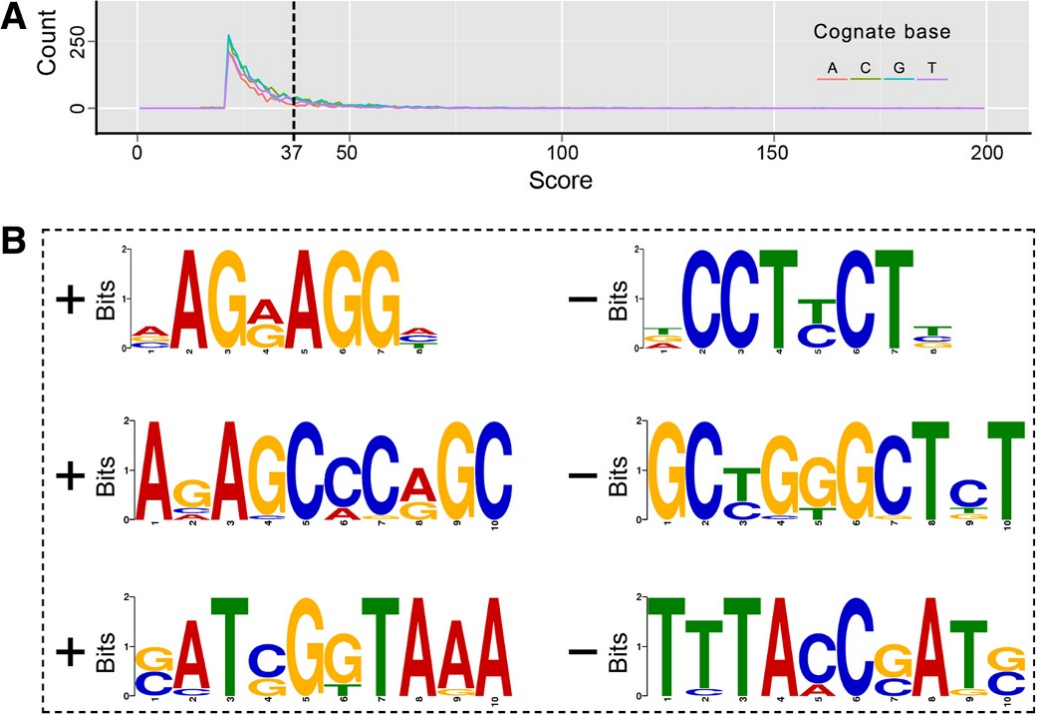
**Modified motif prediction of the PaP1 genome. (A)** Modification scores by cognate base. We selected the top 1,400 context sequences (with a score cutoff of 37) for analysis. A file of 1,400 sequences is suitable as the input for the online motif finding server (MEME). **(B)** Putative modified motifs determined from the PaP1 genome. A left “+” means forward and the corresponding right “–” means reverse complement.

We selected the top 10 modified motifs (with E-value ≤ 5.1e + 004) from numerous motifs screened from the Modifications.gff file and analysed these motifs. We focused on motifs with the number of sites >10; hence, we only acquired three motifs (Figure 5). The consensus sequences of these three motifs are “5′-VAGRAGGH-3′,” “5′-AVASCMSRGC-3′,” and “5′-SMTSGKTARA-3′,” respectively. For these predicted motifs, only some of the sites found in the genome were detected as methylated; this result indicated that the methylated pattern and the methyltransferase (s) PaP1 used may be very complicated.

In silico analysis results revealed that the PaP1 ORF48 is a putative methyltransferase [9]. A total of 15 putative methyltransferases were found when the PaP1 ORF48 was compared with the protein database and the BlastP scores were ≥60 (Table 3). These 15 putative methyltransferases shared 22 identical amino acids (~21%) with the PaP1 ORF48 (Figure 6A). The phylogenetic tree further showed that the PaP1 ORF48 is closely related to the putative methyltransferase encoded by *Pseudomonas* phage JG004 and slightly related to methyltransferases encoded by bacteria (Figure 6B). However, we were unsure whether or not the PaP1 ORF48 is a putative methyltransferase because BlastP analysis results also suggested that the PaP1 ORF48 is related to phage portal proteins.

**Table 3:** **Comparison of PaP1 ORF48 against putative methyltransferases using BlastP**

#	Species	Subject ID	Alignment length	Score	Identity (%)	E-value
1	Pseudomonas phage JG004	gi|418488276|	156	317	99	2e-108
2	*Haliangium ochraceum* DSM 14365	gi|262194136|	137	66	32	3e-10
3	*Lactococcus lactis*	gi|489222890|	141	66	32	5e-10
4	*Lactococcus lactis*	gi|556501605|	128	66	34	6e-10
5	*Lactococcus garvieae*	gi|489228496|	130	66	32	6e-10
6	*Lactococcus lactis* subsp. lactis KLDS 4.0325	gi|554465598|	141	65	32	8e-10
7	*Haliangium ochraceum* DSM 14365	gi|262193963|	137	64	32	3e-09
8	*Enterococcus saccharolyticus*	gi|491614495|	128	62	31	9e-09
9	*Myxococcus xanthus* DK 1622	gi|108757550|	117	61	31	2e-08
10	*Myxococcus xanthus*	gi|521967607|	117	61	31	2e-08
11	*Stigmatella aurantiaca* DW4/3-1	gi|310819901|	117	61	32	3e-08
12	*Myxococcus fulvus* HW-1	gi|338536945|	117	61	32	3e-08
13	*Myxococcus xanthus*	gi|521967482|	117	61	32	3e-08
14	*Myxococcus xanthus*	gi|521967591|	117	61	31	4e-08
15	*Stigmatella aurantiaca* DW4/3-1	gi|310819798|	117	60	32	5e-08

**Figure 6:**
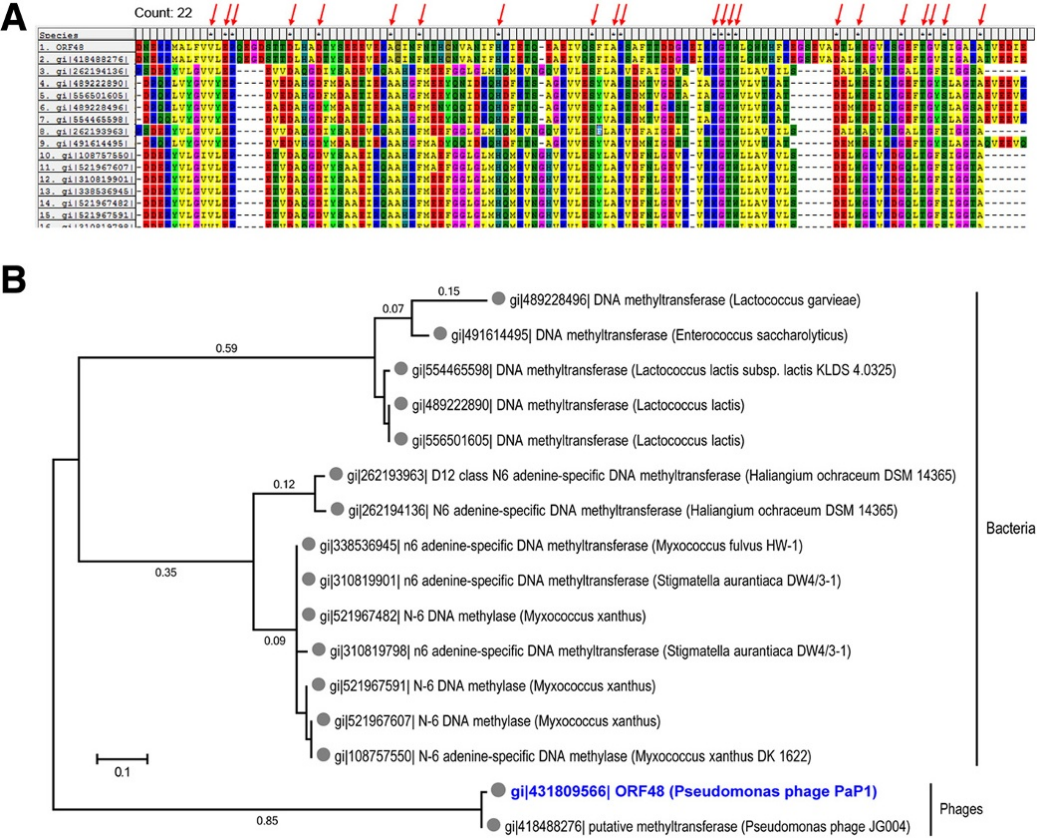
**Relationship of PaP1 ORF48 and related putative methyltransferases. (A)** Multiple sequence alignments of PaP1 ORF48 and related putative methyltransferases (listed in Table 3). **(B)** Phylogenetic analysis of the PaP1 ORF48. This diagram was constructed on the basis of the PaP1 ORF48 and related putative methyltransferases (Table 3). The relative distances of each main branch are also shown in this figure.

### Digestion of the PaP1 genomic DNA by Endo V

**Figure 7:**
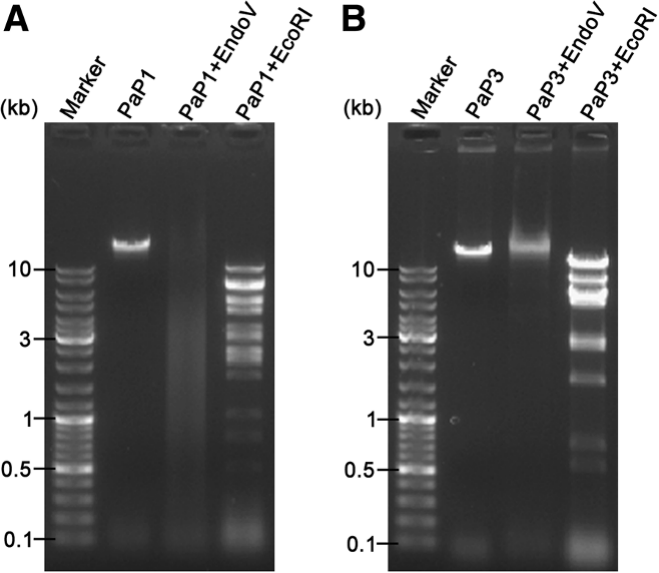
**Agarose gel electropherogram of Endo V digestion. (A)** Digestion of the PaP1 genomic DNA by Endo V or *Eco*RI. Endo V digestion of the PaP1 genomic DNA produced a smear band in the gel. **(B)** Digestion of the PaP3 genomic DNA by Endo V or EcoRI. The PaP3 genome had been successfully sequenced using the shotgun method before. Unlike the PaP1 genomic DNA, Endo V digestion of the PaP3 genomic DNA gave no smear band in the gel.

Some enzymes of the host bacteria (*E. coli* DH5α) of the shotgun library construction probably target these modified bases because the PaP1 genomic DNA contains numerous modified bases. Hence, we doubted enzyme Endo V because this enzyme can recognise and degrade modified bases containing DNA molecules [42–45]. To confirm whether or not Endo V is responsible for the failure of the shotgun method, we used Endo V to digest the genomic DNA of PaP1. The results showed that the PaP1 genomic DNA formed a smear in the gel when this DNA was degraded with Endo V whereas the restriction endonuclease EcoRI cleaved the PaP1 genomic DNA into several independent fragments (Figure 7A). By contrast, the PaP3 genomic DNA [19], successfully sequenced using the shotgun method, cannot be degraded by Endo V under the same reaction condition (Figure 7B); this result suggested that no Endo V cutting site exists in the PaP3 genome.

### Use of the *nfi*^*-*^mutant of *E. coli*DH5α as the host bacterium for shotgun library construction revealed the whole PaP1 genome sequence

**Figure 8:**
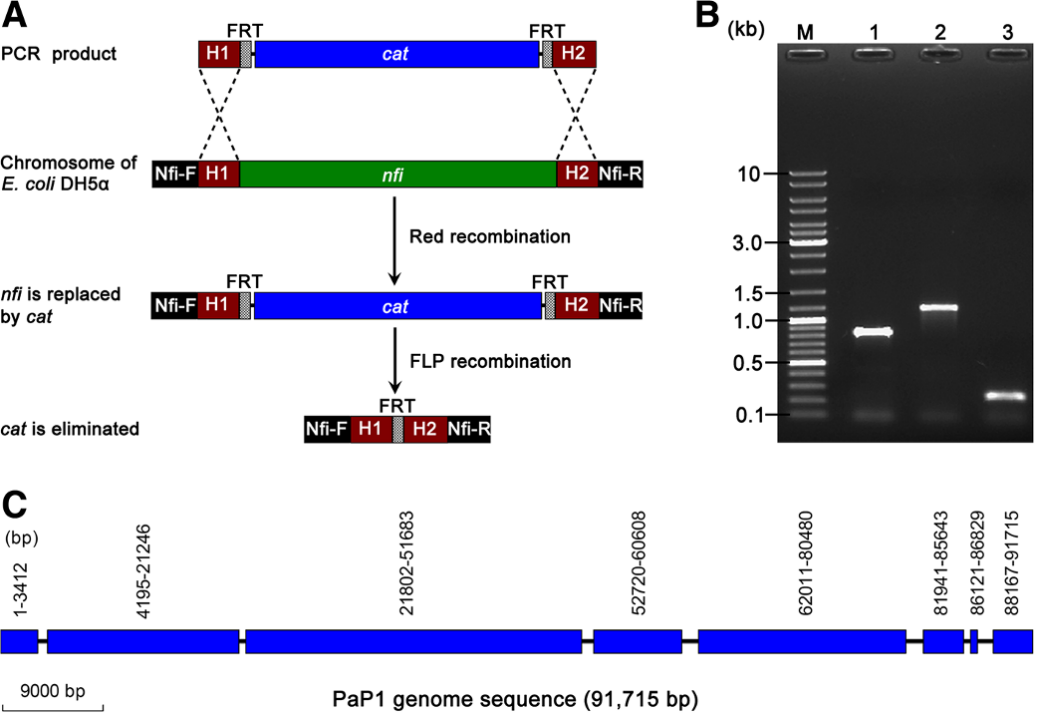
**Construction of the** ***nfi*** ^**-**^ **mutant and its use in shotgun sequencing. (A)** Schematic of the *nfi* gene knockout strategy. The PCR product (donor DNA) containing 55 bp upstream homologous extensions of the *nfi* gene (H1) and 55 bp downstream homologous extensions of the *nfi* gene (H2) was prepared using the pKD3 plasmid as template. The *nfi* gene in the chromosome of *E. coli* DH5α is replaced with chloramphenicol resistant gene (*cat*) by Red recombination of H1 and H2. *cat* is then eliminated by subjecting the FLP recognition target (FRT) sites to FLP recombination; a single FRT site is retained. Nfi-F and Nfi-R are primers indicating the change in the *nfi* gene locus. The length of the region between Nfi-F and Nfi-R primers is 821 bp (*nfi* remain), 1,169 bp (*nfi* is replaced by *cat*), or 237 bp (*cat* is eliminated). **(B)** PCR verification using Nfi-F and Nfi-R primers. Lane 1. Wild-type *E. coli* DH5α (*nfi* remain). Lane 2. *nfi* is replaced with *cat*. Lane 3. *cat* is eliminated. **(C)** Distribution of eight newly obtained contigs in the PaP1 genome. These eight contigs were obtained by shotgun sequencing of the PaP1 genome using *E. coli* DH5α Δ*nfi* as the host to construct shotgun library clones. The blue rectangular boxes represent contigs. The exact location of each contig is indicated by blue boxes.

To further validate the role of Endo V in the failure of the shotgun sequencing of the PaP1 genome and verify the aforementioned hypothesis, we knocked out the Endo V coding gene (*nfi*) of *E. coli* DH5α. The *nfi* gene of *E. coli* DH5α genome was initially substituted with a donor DNA (containing chloramphenicol-resistant gene, *cat*) by using a λ-red recombination system; the *cat* gene was then eliminated by FLP (a yeast-derived recombinase) recombination (Figure 8A). The PCR identification results showed that the sizes of the PCR products are correct (Figure 8B). These PCR products were sequenced and the results indicated that the *nfi* gene was completely knocked out. This mutant was designated as *E. coli* DH5α Δ*nfi* or the *nfi*^-^ mutant of *E. coli* DH5α.

We used this mutant to construct the shotgun library of the PaP1 genomic DNA. The obtained shotgun reads were assembled into eight contigs that covered 92.3% of the PaP1 genome (Figure 8C) when the sequencing coverage reached 10-fold of the PaP1 genome. The length of the largest gap is <1.5 kb, which can be easily filled by primer walking [11]. Hence, the use of *E. coli* DH5α *nfi*^-^ mutant as a host bacterium of shotgun library construction can overcome the inability of the shotgun method to complete the PaP1 genome sequence.

## Discussion

In clone-based genome sequencing, some genomic DNA fragments cannot be cloned using *E. coli*; as a result, cloning gaps are retained when sequence reads are analysed. Although cloning-independent sequencing methods are available [5–7], the cause of the sequencing problem remains unclear. Previous findings indicated that some restriction enzymes [46] and toxic small RNA are present in a shotgun-unclonable genome region. Furthermore, some DNA fragments in shotgun-unclonable regions suppress the growth of *E. coli* [37]. However, the PanDaTox database reveals that the PaP1 genome does not have any evident DNA motifs that are toxic to bacteria; in this study, a different viewpoint was proposed, in which the Endo V-mediated immunity of *E. coli* is responsible for the failure of the shotgun method to sequence a phage genome that contains modified bases.

This study was initiated when we found that the shotgun library method failed to sequence the genome of the PaP1 phage with a size of 90 kb in 2004. Several years later, Roche/454 sequencing method was established. We used the Roche/454 technique to sequence the PaP1 genome again in 2008. We easily obtained the complete genome sequence (91,715 bp) of the PaP1 genome. As such, we wondered why the PaP1 genome could be successfully sequenced using Roche/454 technique but could not be sequenced using the shotgun method. In contrast to the Roche/454 strategy, the shotgun strategy requires shotgun library construction. Based on the principle difference of the two sequencing methods, our presumption was that *E. coli* DH5α, the host bacterium of the shotgun library construction, probably repel the inserted phage-DNA fragments via a particular immune mechanism.

The shotgun strategy has been successfully applied to sequence the genomes of many organisms, including bacteria, plants and animals, as well as viruses. The host bacteria of the constructed shotgun library did not repel the inserted DNA fragments of these organisms. Therefore, the PaP1 genome, as a hard-to-sequence genome, should exhibit a unique characteristic in its genome composition. Considering previous studies, we found that some phage genomes contain modified bases. For instance, deoxycytidines in the genome of Enterobacteria phage T4 are replaced with 5-hydroxymethyldeoxycytidines (5-hmdC) [47, 48]; thymines in the genome of *Bacillus subtilis* phage PBS-1 are substituted by uracils (U) [49]. Thymines in the genomes of *B. subtilis* phage SPO1 [50] and *Delftia acidovorans* pha*ge* ΦW-14 [51, 52] are replaced with 5-hydroxymethyldeoxyuridines (5-hmdU). The phage genomes with modified bases may be commonly observed. These modified bases in a phage genome perform essential functions [53, 54], such as escaping the exclusion of host immune mechanism. During evolution, bacteria most likely develop an immune mechanism that aims directly at these modified bases in exogenous DNA.

Several known bacterial immune mechanisms, such as R-M [55], T-A [56], Abi [57] and CRISPR-Cas [58] systems exist, but any of these mechanisms does not directly aim at varied modified bases in exogenous DNA. We then focused on the enzyme Endo V because this enzyme can recognise many kinds of modified bases in DNA strands [42, 45, 59]. The mechanism of Endo V activity is different from that of general restriction endonucleases in an R-M system because these restriction endonucleases of the R-M system generally recognise and cut at unmodified base sites [60]; by contrast, Endo V recognises and cuts at modified base sites. Endo V also exhibits endonuclease and exonuclease activities [61, 62], which provide Endo V with a more effective DNA destruction activity than general restriction endonucleases.

Endo V was originally reported as a DNA repair enzyme [43, 44, 63] encoded by the *nfi* gene; most bacteria contain the *nfi* gene in their genome. This enzyme can recognise and cleave various modified bases and abnormal structures, such as deaminated bases, abasic (AP) sites, base mismatches, methylated bases, flap DNA, pseudo-Y structures and small insertions/deletions [42, 45, 59, 63] in DNA molecules, with a cleavage site at the second phosphodiester bond in the 3′ direction from the recognition site; as a result, a nick with 5′-phosphate and 3′-hydroxyl groups is formed and DNA strands are greatly disrupted because of the exonuclease activity of this enzyme. To determine whether or not Endo V can destroy the PaP1 genomic DNA, Endo V (a product of *E. coli nfi* gene) was used to digest the PaP1 genomic DNA. The result indicated that Endo V degraded the PaP1 genomic DNA into a smear band (Figure 7A).

To further validate the role of Endo V in the failure of the shotgun sequencing of the PaP1 genome, we knocked out Endo V-coding *nfi* gene and constructed an *nfi*^-^ mutant of *E. coli* DH5α. This mutant was then used as the host bacterium to construct the PaP1 genomic DNA shotgun library. Consequently, the obtained sequences covered 92.3% of the PaP1 genome when the sequencing amount of the PaP1 genome reached a 10-fold coverage and the largest gap between contigs was <1.5 kb (Figure 4), which is very easy to close. This result further confirmed that the activity of Endo V is responsible for the failure of the shotgun sequencing of the PaP1 genome.

The SMRT DNA sequence of the PaP1 genome showed that 7,557 bases of this genome were substituted with modified bases, including 51 m6A, 152 m4C and 7,354 other modified bases (unidentified modified types, Figures 3 and 4). The positions of each modified base in the PaP1 genome (Figure 4) indicated the presence of modified bases in this genome. We also investigated the methylome of the PaP1 phage, which may be the first phage methylome revealed by SMRT technology; this methylome may be significant in future studies on phage biology and host interaction.

## Conclusions

This work revealed the whole PaP1 genome sequence that contains numerous modified bases, provided complete information of the epigenetic information map of the PaP1 phage with 7,557 modified bases and investigated the methylome of PaP1. We found that the shotgun sequencing method is unsuitable for genomes containing many modified bases. To resolve this problem, we may use the *nfi*^-^ mutant of *E. coli* DH5α as the host bacterium of DNA library construction. Moreover, we revealed a new mechanism of bacterial immunity to repel exogenous DNA by Endo V activity. Considering that bacteriophage is a virus infecting bacteria and modified bases are commonly found in a phage genome, the new mechanism of bacterial immunity we first demonstrated in this study, may be particularly necessary for bacteria to evade DNA invasion and retain their genetic stability.

### Availability of supporting data

The nucleotide sequence of PaP1 phage was deposited in the GenBank database with the accession number of HQ832595 (http://www.ncbi.nlm.nih.gov/nuccore/HQ832595). The data sets supporting the results of this article are available in the NCBI GEO repository [34] with the accession number of GSE50100 (http://www.ncbi.nlm.nih.gov/geo/query/acc.cgi?&acc=GSE50100).

## Electronic supplementary material

Additional file 1: Figure S1: Depth of the SMRT sequencing coverage across the PaP1 genome. The window size is set at 200 bp. The average sequencing coverage reached approximately 1,380-fold of the PaP1 genome. (TIFF 359 KB)

Additional file 2: Excel S1: IPD ratios of modified bases within the PaP1 genome, related to Figure 4. This Excel lists the details (including IPD ratios) of 7557 modified bases. (XLSX 457 KB)

## References

[1] Fiers W, Contreras R, Duerinck F, Haegeman G, Iserentant D, Merregaert J, Min Jou W, Molemans F, Raeymaekers A, Van den Berghe A, Volckaert G, Ysebaert M (1976). Complete nucleotide sequence of bacteriophage MS2 RNA: primary and secondary structure of the replicase gene. Nature.

[2] Sanger F, Air GM, Barrell BG, Brown NL, Coulson AR, Fiddes CA, Hutchison CA, Slocombe PM, Smith M (1977). Nucleotide sequence of bacteriophage phi X174 DNA. Nature.

[3] Fleischmann RD, Adams MD, White O, Clayton RA, Kirkness EF, Kerlavage AR, Bult CJ, Tomb JF, Dougherty BA, Merrick JM (1995). Whole-genome random sequencing and assembly of Haemophilus influenzae Rd. Science.

[4] Fuchs TM, Brandt K, Starke M, Rattei T (2011). Shotgun sequencing of Yersinia enterocolitica strain W22703 (biotype 2, serotype O:9): genomic evidence for oscillation between invertebrates and mammals. BMC Genomics.

[5] Gupta PK (2008). Single-molecule DNA sequencing technologies for future genomics research. Trends Biotechnol.

[6] McCarthy A (2010). Third generation DNA sequencing: pacific biosciences’ single molecule real time technology. Chem Biol.

[7] Shendure J, Ji H (2008). Next-generation DNA sequencing. Nat Biotechnol.

[8] Krebes J, Morgan RD, Bunk B, Sproer C, Luong K, Parusel R, Anton BP, Konig C, Josenhans C, Overmann J, Roberts RJ, Korlach J, Suerbaum S (2013). The complex methylome of the human gastric pathogen Helicobacter pylori. Nucleic Acids Res.

[9] Lu S, Le S, Tan Y, Zhu J, Li M, Rao X, Zou L, Li S, Wang J, Jin X, Huang G, Zhang L, Zhao X, Hu F (2013). Genomic and proteomic analyses of the terminally redundant genome of the pseudomonas aeruginosa phage PaP1: establishment of genus PaP1-like phages. PLoS One.

[10] Le S, He XS, Tan YL, Huang GT, Zhang L, Lux R, Shi WY, Hu FQ (2013). Mapping the tail fiber as the receptor binding protein responsible for differential host specificity of pseudomonas aeruginosa bacteriophages PaP1 and JG004. PLoS One.

[11] Benes V, Kilger C, Voss H, Paabo S, Ansorge W (1997). Direct primer walking on P1 plasmid DNA. Biotechniques.

[12] Zheng Z, Advani A, Melefors O, Glavas S, Nordstrom H, Ye W, Engstrand L, Andersson AF (2011). Titration-free 454 sequencing using Y adapters. Nat Protoc.

[13] Clark MS, Thorne MA, Vieira FA, Cardoso JC, Power DM, Peck LS (2010). Insights into shell deposition in the Antarctic bivalve Laternula elliptica: gene discovery in the mantle transcriptome using 454 pyrosequencing. BMC Genomics.

[14] Davis BM, Chao MC, Waldor MK (2013). Entering the era of bacterial epigenomics with single molecule real time DNA sequencing. Curr Opin Microbiol.

[15] Flusberg BA, Webster DR, Lee JH, Travers KJ, Olivares EC, Clark TA, Korlach J, Turner SW (2010). Direct detection of DNA methylation during single-molecule, real-time sequencing. Nat Methods.

[16] Murray IA, Clark TA, Morgan RD, Boitano M, Anton BP, Luong K, Fomenkov A, Turner SW, Korlach J, Roberts RJ (2012). The methylomes of six bacteria. Nucleic Acids Res.

[17] Sambrook J, Russell DW (2006). The Condensed Protocols from Molecular Cloning : A Laboratory Manual.

[18] Sun WZ, Tan YL, Jia M, Hu XM, Rao XC, Hu FQ (2010). Functional characterization of the endolysin gene encoded by Pseudomonas aeruginosa bacteriophage PaP1. Afr J Microbiol Res.

[19] Tan Y, Zhang K, Rao X, Jin X, Huang J, Zhu J, Chen Z, Hu X, Shen X, Wang L, Hu F (2007). Whole genome sequencing of a novel temperate bacteriophage of P. aeruginosa: evidence of tRNA gene mediating integration of the phage genome into the host bacterial chromosome. Cell Microbiol.

[20] Govind R, Fralick JA, Rolfe RD (2006). Genomic organization and molecular characterization of Clostridium difficile bacteriophage PhiCD119. J Bacteriol.

[21] Casas V, Rohwer F (2007). Phage metagenomics. Methods Enzymol.

[22] Vandersteegen K, Kropinski AM, Nash JH, Noben JP, Hermans K, Lavigne R (2013). Romulus and Remus, two phage isolates representing a distinct clade within the Twortlikevirus genus, display suitable properties for phage therapy applications. J Virol.

[23] de la Bastide M, McCombie WR (2007). Assembling genomic DNA sequences with PHRAP. Current protocols in bioinformatics / editoral board, Andreas D Baxevanis [et al].

[24] Chaisson MJ, Pevzner PA (2008). Short read fragment assembly of bacterial genomes. Genome Res.

[25] Cherepanov PP, Wackernagel W (1995). Gene disruption in Escherichia coli: TcR and KmR cassettes with the option of Flp-catalyzed excision of the antibiotic-resistance determinant. Gene.

[26] Datsenko KA, Wanner BL (2000). One-step inactivation of chromosomal genes in Escherichia coli K-12 using PCR products. Proc Natl Acad Sci U S A.

[27] Powers JG, Weigman VJ, Shu J, Pufky JM, Cox D, Hurban P (2013). Efficient and accurate whole genome assembly and methylome profiling of E. coli. BMC Genomics.

[28] Wittmann J, Dreiseikelmann B, Rohde M, Meier-Kolthoff JP, Bunk B, Rohde C (2014). First genome sequences of Achromobacter phages reveal new members of the N4 family. Virol J.

[29] Clark TA, Murray IA, Morgan RD, Kislyuk AO, Spittle KE, Boitano M, Fomenkov A, Roberts RJ, Korlach J (2012). Characterization of DNA methyltransferase specificities using single-molecule, real-time DNA sequencing. Nucleic Acids Res.

[30] Travers KJ, Chin CS, Rank DR, Eid JS, Turner SW (2010). A flexible and efficient template format for circular consensus sequencing and SNP detection. Nucleic Acids Res.

[31] Levene MJ, Korlach J, Turner SW, Foquet M, Craighead HG, Webb WW (2003). Zero-mode waveguides for single-molecule analysis at high concentrations. Science.

[32] Chaisson MJ, Tesler G (2012). Mapping single molecule sequencing reads using basic local alignment with successive refinement (BLASR): application and theory. BMC Bioinformatics.

[33] Lluch-Senar M, Luong K, Llorens-Rico V, Delgado J, Fang G, Spittle K, Clark TA, Schadt E, Turner SW, Korlach J, Serrano L (2013). Comprehensive Methylome Characterization of Mycoplasma Genitalium and Mycoplasma Pneumoniae at Single-Base Resolution. PLoS Genet.

[34] Barrett T, Wilhite SE, Ledoux P, Evangelista C, Kim IF, Tomashevsky M, Marshall KA, Phillippy KH, Sherman PM, Holko M, Yefanov A, Lee H, Zhang N, Robertson CL, Serova N, Davis S, Soboleva A (2013). NCBI GEO: archive for functional genomics data sets–update. Nucleic Acids Res.

[35] Rosseel T, Scheuch M, Hoper D, De Regge N, Caij AB, Vandenbussche F, Van Borm S (2012). DNase SISPA-next generation sequencing confirms Schmallenberg virus in Belgian field samples and identifies genetic variation in Europe. PLoS One.

[36] Schattner P, Brooks AN, Lowe TM (2005). The tRNAscan-SE, snoscan and snoGPS web servers for the detection of tRNAs and snoRNAs. Nucleic Acids Res.

[37] Kimelman A, Levy A, Sberro H, Kidron S, Leavitt A, Amitai G, Yoder-Himes DR, Wurtzel O, Zhu YW, Rubin EM, Sorek R (2012). A vast collection of microbial genes that are toxic to bacteria. Genome Res.

[38] Bailey TL, Boden M, Buske FA, Frith M, Grant CE, Clementi L, Ren J, Li WW, Noble WS (2009). MEME SUITE: tools for motif discovery and searching. Nucleic Acids Res.

[39] Chenna R, Sugawara H, Koike T, Lopez R, Gibson TJ, Higgins DG, Thompson JD (2003). Multiple sequence alignment with the Clustal series of programs. Nucleic Acids Res.

[40] Tamura K, Peterson D, Peterson N, Stecher G, Nei M, Kumar S (2011). MEGA5: molecular evolutionary genetics analysis using maximum likelihood, evolutionary distance, and maximum parsimony methods. Mol Biol Evol.

[41] Som A, Fuellen G (2009). The effect of heterotachy in multigene analysis using the neighbor joining method. Mol Phylogenet Evol.

[42] Dalhus B, Arvai AS, Rosnes I, Olsen OE, Backe PH, Alseth I, Gao H, Cao W, Tainer JA, Bjoras M (2009). Structures of endonuclease V with DNA reveal initiation of deaminated adenine repair. Nat Struct Mol Biol.

[43] Gates FT, Linn S (1977). Endonuclease V of Escherichia coli. J Biol Chem.

[44] Liu J, He B, Qing H, Kow YW (2000). A deoxyinosine specific endonuclease from hyperthermophile, Archaeoglobus fulgidus: a homolog of Escherichia coli endonuclease V. Mutat Res.

[45] Weiss B (2001). Endonuclease V of Escherichia coli prevents mutations from nitrosative deamination during nitrate/nitrite respiration. Mutat Res.

[46] Zheng Y, Posfai J, Morgan RD, Vincze T, Roberts RJ (2009). Using shotgun sequence data to find active restriction enzyme genes. Nucleic Acids Res.

[47] Childs JD, Ellison MJ, Pilon R (1983). Formation of 5-hydroxymethylcytosine-containing pyrimidine dimers in UV-irradiated bacteriophage T4 DNA. Photochem Photobiol.

[48] Lehman IR, Pratt EA (1960). On the structure of the glucosylated hydroxymethylcytosine nucleotides of coliphages T2, T4, and T6. J Biol Chem.

[49] Takahashi I, Marmur J (1963). Replacement of thymidylic acid by deoxyuridylic acid in the deoxyribonucleic acid of a transducing phage for Bacillus subtilis. Nature.

[50] Kallen RG, Simon M, Marmur J (1962). The new occurrence of a new pyrimidine base replacing thymine in a bacteriophage DNA:5-hydroxymethyl uracil. J Mol Biol.

[51] Kropinski AM, Bose RJ, Warren RA (1973). 5-(4-Aminobutylaminomethyl)uracil, an unusual pyrimidine from the deoxyribonucleic acid of bacteriophage phiW-14. Biochemistry.

[52] Maltman KL, Neuhard J, Warren RA (1981). 5-[(Hydroxymethyl)-O-pyrophosphoryl]uracil, an intermediate in the biosynthesis of alpha-putrescinylthymine in deoxyribonucleic acid of bacteriophage phi W-14. Biochemistry.

[53] Gommers-Ampt JH, Borst P (1995). Hypermodified bases in DNA. FASEB J.

[54] Warren RA (1980). Modified bases in bacteriophage DNAs. Annu Rev Microbiol.

[55] Xu SY, Nugent RL, Kasamkattil J, Fomenkov A, Gupta Y, Aggarwal A, Wang X, Li Z, Zheng Y, Morgan R (2012). Characterization of type II and III restriction-modification systems from Bacillus cereus strains ATCC 10987 and ATCC 14579. J Bacteriol.

[56] Fineran PC, Blower TR, Foulds IJ, Humphreys DP, Lilley KS, Salmond GP (2009). The phage abortive infection system, ToxIN, functions as a protein-RNA toxin-antitoxin pair. Proc Natl Acad Sci U S A.

[57] Friedman DI, Mozola CC, Beeri K, Ko CC, Reynolds JL (2011). Activation of a prophage-encoded tyrosine kinase by a heterologous infecting phage results in a self-inflicted abortive infection. Mol Microbiol.

[58] Datsenko KA, Pougach K, Tikhonov A, Wanner BL, Severinov K, Semenova E (2012). Molecular memory of prior infections activates the CRISPR/Cas adaptive bacterial immunity system. Nat Commun.

[59] Childs JD, Paterson MC, Smith BP, Gentner NE (1978). Evidence for a near UV-induced photoproduct of 5-hydroxymethylcytosine in bacteriophage T4 that can be recognized by endonuclease V. Mol Gen Genet.

[60] Bickle TA, Kruger DH (1993). Biology of DNA restriction. Microbiol Rev.

[61] Huang J, Lu J, Barany F, Cao W (2001). Multiple cleavage activities of endonuclease V from Thermotoga maritima: recognition and strand nicking mechanism. Biochemistry.

[62] Majorek KA, Bujnicki JM (2009). Modeling of Escherichia coli Endonuclease V structure in complex with DNA. J Mol Model.

[63] Rosnes I, Rowe AD, Vik ES, Forstrom RJ, Alseth I, Bjoras M, Dalhus B (2013). Structural basis of DNA loop recognition by endonuclease V. Structure.

