# Supplementary figures and images for: Unlocking the mystery of the hard-to-sequence phage genome: PaP1 methylome and bacterial immunity
Source: BMC Genomics. 2014 Sep 19;15(1):803. doi: 10.1186/1471-2164-15-803 (PMC4177049; doi:10.1186/1471-2164-15-803)

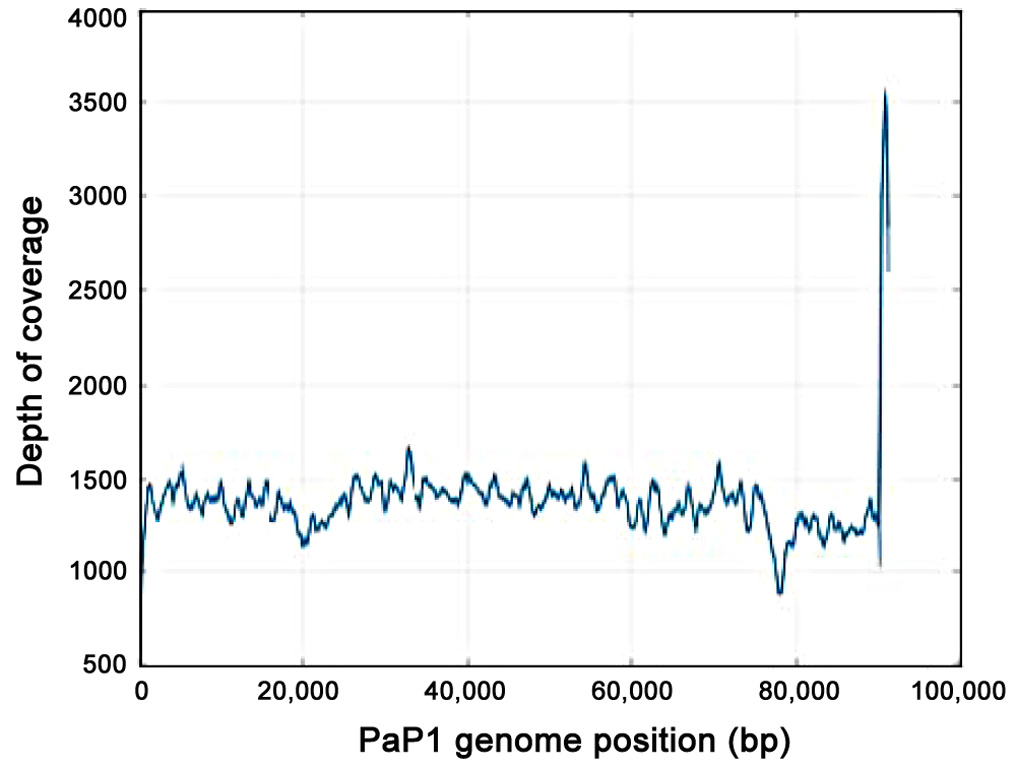

Supplement: Supplementary file 1 — Additional file 1: Figure S1: Depth of the SMRT sequencing coverage across the PaP1 genome. The window size is set at 200 bp. The average sequencing coverage reached approximately 1,380-fold of the PaP1 genome. (TIFF 359 KB) [file 12864_2014_6479_MOESM1_ESM.tiff]
